# Suppressive effect of goat bile in *Plasmodium berghei* ANKA infection in mice

**DOI:** 10.14202/vetworld.2021.2016-2022

**Published:** 2021-08-06

**Authors:** Heny Arwati, Ramadhani R. Bahalwan, Windya T. Hapsari, Kartika A. Wardhani, Kholida N. Aini, Putu I. B. Apsari, Puspa Wardhani

**Affiliations:** 1 Department of Medical Parasitology, Faculty of Medicine, Universitas Airlangga, Campus A, Jl. Prof. Dr. Moestopo No. 47, Surabaya 60131, Indonesia; 2 Department of Medical Pharmacology, Faculty of Medicine, Universitas Airlangga, Campus A, Jl. Prof. Dr. Moestopo No. 47, Surabaya 60131, Indonesia; 3 Department of Opthalmology, Dr. Soetomo Hospital, Jl. Prof. Dr. Moestopo No. 6-8, Surabaya 60286, Indonesia; 4 Master Program on Immunology, Postgraduate School, Universitas Airlangga, Campus B, Jl. Darmawangsa Dalam Selatan No. 30, Surabaya 60286, Indonesia; 5 Department of Immunology and Microbiology, Karya Putra Bangsa Institute of Health Science, Jalan Raya Tulungagung-Blitar Km 4, Tulungagung 66291, Indonesia; 6 Department of Microbiology and Parasitology, Faculty of Medicine, Universitas Marwadewa, Jl. Terompong No.24, Denpasar, Bali 80235, Indonesia; 7 Department of Clinical Pathology, Faculty of Medicine, Universitas Airlangga, Campus A, Jl. Prof. Dr. Moestopo No. 47, Surabaya 60131, Indonesia; 8 Department of Clinical Pathology, Dr. Soetomo Hospital, Jl. Prof. Dr. Moestopo No. 6-8, Surabaya 60286, Indonesia

**Keywords:** blood biochemistry, goat bile, hepatomegaly, malaria, splenomegaly, suppressive effect

## Abstract

**Background and Aim:**

Some individuals in Indonesia consume intact goat gallbladder to prevent and treat malaria. The acute and subacute toxicity tests of goat bile (GB) have shown mild diarrhea in mice. Therefore, this study aimed to evaluate the suppressive effect of GB on parasitemia, splenomegaly, hepatomegaly, and blood biochemistry to assess liver and kidney function in BALB/c mice infected with *Plasmodium* *berghei* ANKA.

**Materials and Methods:**

Fifty healthy mice were infected with *P. berghei* ANKA and divided into five groups. Mice in three groups were administered 0.5 mL of 25%, 50%, or 100% of GB by gavage. Animals in Group 4 were administered 187.2 mg/kg BW of dihydroartemisinin-piperaquine phosphate as a positive control (POS Group). Mice in fifth group were administered sterile water as negative (NEG) controls. Further, 30 uninfected mice were divided into groups 6-8 and administered GB as were mice in the first three groups. Group 9 included 10 uninfected and untreated animals as healthy controls. Treatments were administered in a 4-day suppressive test followed by daily observation of Giemsa-stained blood smears. On day 7, mice were sacrificed to measure the length and weight of spleens and livers, plasma levels of aspartate aminotransferase (AST), alanine aminotransferase (ALT), blood urea nitrogen (BUN), and creatinine.

**Results:**

GB suppressed parasitemia but did not affect the size and weight of spleens or livers or plasma levels of AST and ALT compared to uninfected GB-treated and healthy control animals. Conversely, plasma levels of BUN and creatinine were suppressed and remained in the normal range in all groups of mice.

**Conclusion:**

GB suppresses parasitemia with no significant impact on hepatic enzymes in GB-treated infected mice. Liver dysfunction in GB-treated infected mice was due to *P. berghei* rather than GB treatment.

## Introduction

Malaria is a parasitic infection caused by protozoa in the genus *Plasmodium*. Human malaria is caused by four *Plasmodium* species, *Plasmodium* *falciparum*, *Plasmodium vivax*, *Plasmodium ovale*, and *Plasmodium* *malariae* [1]. *Plasmodium* *knowlesi* is a fifth human parasite [2] after transmission from Macaque was identified in Borneo, Malaysia [3]. Globally 229 million malaria cases were estimated in 2019 in 87 countries where malaria is endemic. This number declined from an estimated 238 million in 2000. Twenty-nine of these 87 countries account for 95% of malaria cases globally [4]. Malaria eradication in Indonesia reached the halfway point in 2018, largely due to accelerated progress in the past decade [5]. Annual parasite incidence showed a national decline from 1.8 in 2009 to 0.84 in 2019. However, a small increase to 0.93 was reported in 2019, even though nationally 300 districts/cities were declared malaria-free. This number increased from 285 districts/cities declared malaria-free in 2018 [6]. Other issues, such as asymptomatic and submicroscopic malaria in some areas of Indonesia, have not been addressed [7-9]. Malaria control in Indonesia still faces the emergence of parasite resistance to antimalarial drugs, such as chloroquine (CQ), sulfadoxine-pyrimethamine, and artemisinin combination therapy [10]. Alternatively, traditional medicines from natural ingredients, such as herb extracts, are being explored. In Indonesia, some individuals consume intact goat gallbladder to prevent and treat malaria [11]. Goat meat is commonly consumed and gallbladder is readily available in slaughterhouses or during Eid Al-Adha, an Islamic celebration day. Gallbladder is not consumed due to its bitter taste.

The gallbladder is a small organ that stores bile. Bile is a digestive fluid secreted by hepatocytes. Bile contains water and electrolytes, organic compounds, such as bile salts, cholesterol, phospholipids, bilirubin, and ingested compounds, such as proteins. Bile acids are important for the digestion and absorption of fats and fat-soluble vitamins from the small intestine [12,13]. Animal bile has been used in traditional Chinese medicine (TCM) for centuries to treat chronic and acute infectious and non-infectious diseases, including malaria. Bile supports liver function, dissolves gallstones, and inhibits bacterial and viral multiplication. Bile also exhibits anti-inflammatory, anti-pyretic, and anti-oxidant properties [14]. Bear bile is used to treat liver disease [15]; however, no history is available for the use of goat bile (GB) to treat and cure malaria.

The acute and subacute toxicity tests of GB have shown mild diarrhea in BALB/c mice [11]. Therefore, this study aimed to evaluate the suppressive effect of GB on parasitemia, splenomegaly, hepatomegaly, and blood biochemistry in mice infected with *Plasmodium berghei* ANKA.

## Materials and Methods

### Ethical approval

The study was approved by the Ethics Committee of Faculty of Medicine, Universitas Airlangga (No. 195/EC/KEPK/FKUA/2018).

### Study period and location

The study was conducted from May to July 2018 in the Faculty of Medicine, Universitas Airlangga. Malaria infection in mice was done in the Experimental Animal Laboratory. Determination of parasitemia was done in the Department of Medical Parasitology. Measurement of blood biochemistry was performed in the Department of Clinical Pathology of Dr. Soetomo Hospital.

### Preparation of GB

Goat gallbladders were from a local animal slaughterhouse in Surabaya, East Java Province. The goat Java strain is most raised in Surabaya. Goat gallbladders were isolated from seven healthy male goats. Gallbladders were sprayed with 70% alcohol; then bile was removed by syringe, transferred, and pooled in a clean tube, and diluted with distilled water to prepare 100% (GB100), 50% (GB50), and 25% (GB25) [11]. Samples were stored at 4°C before and during experiments.

### Suppressive test

The study was a 4-day suppression test [16]. Healthy male mice aged ±7 weeks and weighing ±25 g were active and without morphological abnormalities (inclusion criteria). Mice that died during the experiment were excluded (exclusion criteria). The same inclusion and exclusion criteria were applied to mice in positive and negative control groups. *P. berghei* ANKA-infected blood was obtained from donor mice that showed parasitemia levels of 15-20%. Inocula were prepared based on percentage parasitemia and number of erythrocytes counted using a Neubeaur hemocytometer. After 1 week of acclimatization, 50 mice were injected with 1×10^6^ *P.berghei* ANKA-infected erythrocytes in 0.2 mL of blood suspension. Mice were then divided randomly into five groups prior to treatment. Three groups –GB25, GB50, and GB100 –were administered GB25, GB50, and GB100, respectively. Group 4 was a positive control (POS) administered 187.2 mg/kg BW of dihydroartemisinin-piperaquine phosphate (DHP) (Mersipharma, Sukabumi, Indonesia). Group 5 was a negative control (NEG) administered sterile water. Simultaneously, 30 uninfected mice were divided into three groups –NOR25, NOR50, and NOR100 –and treated with GB25, GB50, and GB100, respectively. In addition, ten uninfected mice were treated with sterile water and maintained as the normal (NOR) group. GB treatment was administered in 0.5 mL of GB/25 g BW per day for four consecutive days, followed by observation of parasitemia on Giemsa-stained thin tail blood films. Percent parasitemia and percent suppression were calculated as per formula [17]:


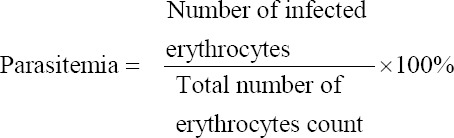



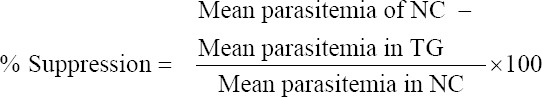


NC: Negative control; TG: Treated Group

### Splenomegaly and hepatomegaly

On day 7, mice were sacrificed after induction of general anesthesia by chloroform inhalation. Spleens and livers were removed and weight and length measured to assess splenomegaly and hepatomegaly. Length was measured with a ruler and weight with an analytical scale [18]. Plasma was collected for hepatic and renal enzyme evaluation.

### Liver and kidney function

Liver and kidney function was assessed using plasma levels of aspartate aminotransferase (AST) and alanine aminotransferase (ALT) activity, blood urea nitrogen (BUN), and creatinine using an automatic hematology analyzer. Blood biochemistry analysis was performed in the Department of Clinical Pathology, Dr. Soetomo Hospital/Faculty of Medicine, Universitas Airlangga using standard operating procedures for daily laboratory activities. Fifty microliters of plasma were used for analysis of AST, ALT, BUN, and creatinine.

### Statistical analysis

Data were entered into a Microsoft Excel spreadsheet, exported, and analyzed using SPSS version 20 (IBM Corp., NY, USA). Estimation of survival time of mice was analyzed using Kaplan–Meier test. Comparisons among treatment, negative and positive control mice were assessed using one-way analysis of variance (ANOVA) for normally distributed data. When ANOVA showed a significant difference, *post hoc* Bonferroni, or Games Howell multiple comparison tests were used to assess differences among groups. The Mann–Whitney U-test was used to assess differences when data were not normally distributed. Correlations among parasitemia and splenomegaly, hepatomegaly, and blood biochemistry were analyzed using Pearson or Spearman correlation tests depending on data distribution. Differences were considered statistically significant at a 95% confidence level (p<0.05).

## Results

### Toxicity and survival rate

**Figure-1:**
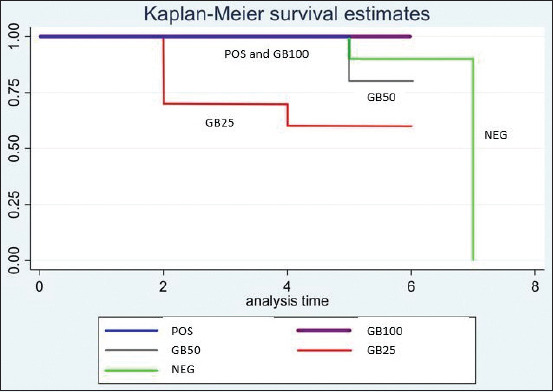
Kaplan-Meier survival estimate of *Plasmodium* *berghei* ANKA-infected mice treated with goat bile (GB) compared with controls. All mice in GB100 were survived and gave 100% survival rate as those in POS (blue and purple lines are overlapping). Two mice in GB50 died on day 5 post-treatment and gave 80% survival rate. Two mice of GB25 died at day 2, other two 2 mice died on day 4, resulted in 60% survival rate, while in NEG one mouse died on day 5 and all mice died on day 7.

GB caused mild diarrhea in non-infected animals [11]. Diarrhea also was seen in some, but not all, *P. berghei* ANKA-infected-mice treated with GB in the present study. The highest dose of GB (GB100) caused diarrhea in the largest number of mice (Table-1). The survival rate of infected-mice treated with GB is also shown in Table-1. Kaplan–Meier estimation of survival time is summarized in Figure-1. Survival rate of mice in GB100 was 100%, as same as in POS. Two mice in GB50 died on day 5 post-treatment and gave 80% survival rate. Two mice of GB25 died on day 2, other 2 mice died on day 4, resulted in 60% survival rate, while in NEG one mouse died on day 5 and all mice died on day 7 (0% survival rate).

**Table 1:** Number of mice with diarrhea, survival rate, parasitemia, and suppressive effect of GB in mice infected with *Plasmodium berghei* ANKA.

Group of treatment	Number of mice with diarrhea (%)*	Survival rate of mice (%)*	Parasitemia±SD (%)**	Suppression (%)**
GB25	2 (20)	60	19.52±3.58	42.99
GB50	1 (10)	80	4.48±2.40	86.92
GB100	8 (80)	100	1.06±0.88	96.90
POS	0 (0)	100	0.04±0.01	99.88
NEG	0 (0)	0	34.24±1.34	

* n=10,

** n=5, at day 6 post-treatment with GB. GB: Goat bile

### Suppressive effect of GB

**Figure-2:**
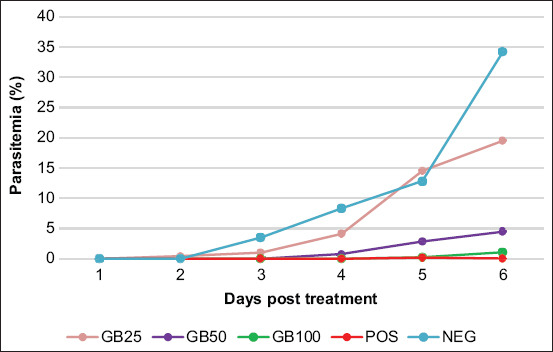
Parasitemia in Plasmodium berghei ANKA-infected mice treated with goat bile (GB): GB25, GB50, and GB100 in a four- day suppressive test compared with that of POS (DHP) and NEG (water).

Oral administration of 25%, 50%, and 100% of GB suppressed parasitemia in a dose-dependent manner by 42.99%, 86.92%, and 96.90%, respectively (Table-1). Animals that received GB showed reduced parasitemia (Figure-2). Observation of parasitemia continued for 2 days post-treatment. Daily parasitemia in negative control mice (NEG) developed in a typical fashion and increased sharply to 34.24% on day 6 post-treatment. Parasitemia in GB25-treated mice was similar. Parasites appeared on day 2 post-treatment and reached 19.52% (p=0.000) parasitemia in GB50-treated mice appeared on day 3 post-treatment and reached 4.48% (p=0.000). GB100 treated mice showed suppression similar to mice treated with DHP. Parasitemia reached 1.06% (p=0.030). DHP-treated animals (POS) showed complete suppression of parasitemia on day 4; however, a few erythrocytes were infected by day 5 and parasitemia reached 0.04% on day 6. DHP treatment reduced infection by 99.88% (p=0.043) (Table-1, Figure-2).

### Splenomegaly and hepatomegaly

**Figure-3:**
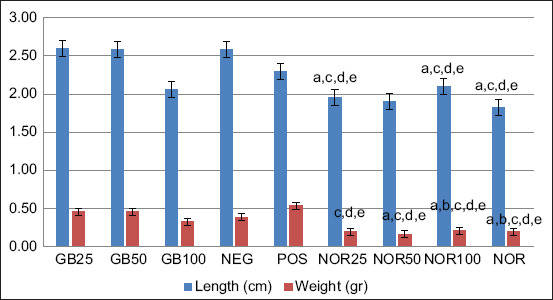
The length and weight of spleen of goat bile (GB)-treated *Plasmodium berghei* ANKA-infected mice (GB25, GB50, and GB100) compared with those of GB-treated uninfected mice (NOR25, NOR50, and NOR100), untreated normal mice (NOR), and DHP-treated (POS) and untreated-infected mice (NEG). The following codes are significantly different (p<0.05) with: a. POS, b. NEG, c. GB25, d. GB50, e. GB100. Without code: non-significantly different (p>0.05).

The appearance of spleen and liver in infected-mice treated with GB, DHP (POS), and in untreated animals (NEG) was similar. However, a significant difference was seen in length of spleen in POS animals compared with NOR25 (uninfected) (p=0.035), NOR50 (p=0.028), NOR100 (p=0.028), and NOR (p=0.033) animals. Spleen weight in POS mice was significantly different from weights in NOR50 (p=0.032), NOR100 (p=0.019), and in NOR (p=0.015) mice. Comparison with NEG mice, only the weight of spleens from GB100-treated uninfected mice (NOR100) and NOR was significantly different (p=0.037 and p=0.033, respectively); but were not significantly different from DHP-treated mice (POS). Interestingly, the length and weight of spleen and liver of GB25, GB50, and GB100-treated animals were significantly different from organs of NOR25, NOR50, NOR100, and NOR mice (p=0.009-0.047). No significant differences in length and weight of spleen or liver among NOR25, NOR50, NOR100 animals were observed, except for liver length in NOR50 animals compared to NOR25 (p=0.046) and NOR100 (p=0.046) mice (Figure-3). Further, correlations between parasitemia and lengths and weights of spleen and liver were found only for spleen weights (p=0.046), a positive correlation was observed between length and weight of spleen (p=0.000).

### Liver and kidney function

Plasma levels of ALT and AST activity from infected-mice treated with GB and untreated mice (NEG) were much higher compared with levels of untreated normal mice (NOR) and uninfected mice treated with GB (NOR25. NOR50, NOR100). The latter results were within the normal range [19]. ANOVA and *post hoc* Tukey analysis resulted for all GB-treated mice (GB25, GB50, and GB100), POS, and untreated infected mice (NEG) showed a significant difference in AST (p=0.000-0.001) compared with those of NOR25, NOR50, NOR100, and NOR animals, and with POS mice (Table-2) [19]. Plasma levels of ALT in GB50 and GB100 were, respectively, significantly different when compared with that of POS animals (p=0.007 and 0.011), NOR25 (p=0.004 and 0.002), NOR50 (p=0.004 and 0.0.002), NOR100 (p=0.002 and 0.001), and NOR (p=0.004 and 0.002). However, significant differences were not observed in comparison with NEG animals (p=0.603). Plasma levels of ALT and AST among GB-treated uninfected (NOR25, NOR50, and NOR100) and untreated (NOR) mice were not significantly different. Levels of ALT, AST, BUN, and creatinine in all infected animals in GB25, GB50, GB100, POS, and NEG animals were not correlated with parasitemia (p>0.05).

**Table 2:** Plasma level of ALT, AST, BUN, and creatinine of *Plasmodium berghei* ANKA-infected, uninfected, and normal mice treated and untreated with GB25, GB50, and GB100 compared with those of POS and NEG groups.

Group of mice	ALT	AST	BUN	Creatinine
GB25	619.2±188.23	1261.6±118.8^a,b^	18.4±0.89	0.23±0.07
GB50	599.8±82.27^a^	210.4±96.86^a,b^	29.4±11.01	0.332±0.14
GB100	581.8±233.73^a^	257.4±66.68^a,b^	28.6±8.62	0.204±0.11
NEG	511.8±91.36	141.0±79.69^a,b^	25.8±3.89	0.188±0.06
POS	170±58.70	43.20±13.23	15.6±1.67	0.182±0.04
NOR25	104.4±28.23	46.0±16.69	16.8±1.92	0.098±0.07
NOR50	103.2±17.94	46.0±6.52	20.8±5.45	0.124±0.017
NOR100	119±7.84	33.8±7.29	17.8±2.59	0.088±0.05
NOR	95.8±5.22	43.4±4.04	17.24±0.78	0.156±0.02
[19]	54-298	17-77	8.0-33	0.2-0.9

The gray shaded-area is plasma levels of ALT, AST that exceeded normal levels [19]. The following codes are significantly different (p<0.05) with: a. POS, NOR25, NOR50, NOR100, and NOR; b. NEG. Without codes are non-significantly different (p>0.05). AST: Aspartate aminotransferase, ALT: Alanine aminotransferase, BUN: Blood urea nitrogen, GB: Goat bile

## Discussion

The antimalarial activity of GB is verified [20]. GB treatment caused mild diarrhea within 2 days post-treatment in uninfected mice [11]. This same response also occurred in *P. berghei* ANKA-infected mice treated with GB [20]. Normally, excess bile acid in the human colon causes classic signs and symptoms of bile acid malabsorption, including watery stool, increased bowel frequency, urgency, nocturnal defecation, excessive flatulence, abdominal pain, and stool incontinence [21]. Oral treatment of mice with GB also caused malabsorption of bile acids and mild to watery diarrhea. Other symptoms, such as the frequency of bowel movements, were not recorded. No formal report of diarrhea in humans caused by consumption of whole goat gall bladder to treat malaria or increase stamina is available.

Parasitemia in POS mice was almost totally suppressed by the administration of the antimalarial drug, DHP, showing that the drug is an appropriate positive control [22]. Parasitemia in GB100-treated mice was somewhat higher than levels in these control animals but significantly lower than levels in untreated NEG mice. Such efficacy was previously reported [22]. GB100 treatment showed significant suppression of parasitemia (96.88%), suggesting efficacy similar to DHP. Lower concentrations of GB were less effective, indicating a dose-response relationship. Survival of *Plasmodium* infected-mice was also proportional to GB dose. Higher concentrations of GB were associated with increased survival. Survival of infected-mice treated with GB100 was 100%, as same as seen for mice treated with DHP. However, GB100 administration caused a higher incidence of diarrhea (eight mice). GB50 administration caused diarrhea in only one mouse. A dose equivalent to GB50 might provide a significant reduction in parasitemia (86.92%) with lower toxicity. Observed antimalarial activity of GB is consistent with the traditional use of GB to treat malaria by indigenous people in Indonesia, where the whole gallbladder is consumed directly without any preparation (100% GB) [11].

The mechanism of action of GB in suppressing parasite multiplication in erythrocytes needs further investigation. This activity might reflect the complexity of bile components, such as amphipathic properties of bile acids that are associated with both benefits and toxicity. Hydrophilic bile acids, ursodeoxycholic acid (UDCA), and tauroursodeoxycholic acid, help repair damage and protect against the toxicity of hydrophobic bile acid, deoxycholic acid [11]. The high pH of bile may create an alkaline condition in the parasite acidic food vacuole. Alkalinization of the vacuole is the mechanism of CQ action in killing malaria parasites [23], susceptibility of malaria parasites to CQ in humans is pH dependent [24]. Accumulation of CQ in vacuoles is governed by the weak basic property of CQ [23,24]. Malaria parasite consumes hemoglobin in erythrocytes and digests this food in acidic vacuoles. Changes in pH inhibit breakdown of hemoglobin by cysteinase and thus starve parasite.

**Figure-4:**
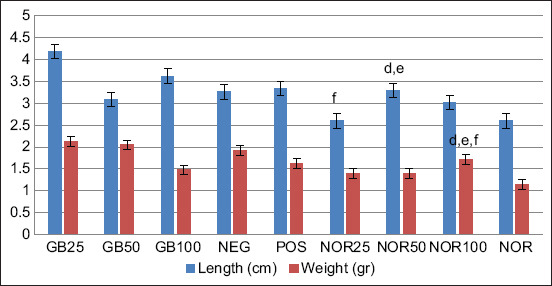
The length and weight of liver of goat bile (GB)-treated infected mice (GB25, GB50, and GB100) compared with those of GB-treated uninfected mice (NOR25, NOR50, and NOR100) and GB-untreated normal mice (NOR). The following codes are different significantly (p<0.05) with: a. POS, b. NEG, c. GB25, d. GB50, e. GB100, f. NOR25. Without code: non-significantly different (p>0.05).

GB treatment generally showed no significant effect on splenomegaly or hepatomegaly in *P. berghei* ANKA-infected mice; however, length and weight of spleen and liver were significantly different when compared with either uninfected mice treated with GB (NOR25, NOR50, and NOR100) or untreated uninfected mice (NOR). GB treatment thus did not affect spleen or liver size in uninfected mice. Thus, enlargement of these organs in infected mice was caused by malarial parasites (Figures-3 and 4). Further, statistically, no significant correlation between length and weight of spleen or liver with parasitemia was observed (p>0.05).

Plasma levels of ALT and AST in *P. berghei* ANKA-infected-mice treated with GB (GB25, GB50, and GB100) and untreated mice (NEG) were much higher than activities in DHP-treated (POS), uninfected mice, and normal mice treated with GB (NOR25, NOR50, NOR100, and NOR). The latter mice showed enzyme activities in the normal range [18]. Elevated ALT and AST of *P. berghei* ANKA-infected mice indicate that DHP is a potent drug that suppresses the growth of parasites and restores plasma ALT and AST levels to normal. Furthermore, high plasma hepatic enzyme activities were not caused by GB treatment. Instead, malarial infection caused enzyme release from the liver. ALT and AST levels remained normal in GB-treated and untreated uninfected mice (NOR25, Nor50, NOR100, and NOR).

Degenerative changes in hepatocytes caused by malaria parasites may cause hepatic dysfunction, a common feature of severe malaria [25]. This condition is characterized by significant increases in liver enzyme activities, such as plasma ALT, AST, and alkaline phosphatase in plasma [26]. GB treatment did not affect plasma enzymes in GB-treated infected mice.

Conversely, kidney function in all mice remained normal, as plasma levels of BUN and creatinine remained in the normal range [19]. Plasma levels of BUN and creatinine in treated uninfected and untreated mice also remained normal. Thus, GB treatment did not adversely affect kidney function. Consumption of fish gall bladder caused acute renal failure (ARF) in India [27], China [28], Vietnam [29], Cambodia [30], and, rarely, in Japan [31]. In Indonesia, Java Barb fish gallbladder caused both acute renal and hepatic injury [32]. Fish gallbladder is consumed to improve vision and treat rheumatism and asthma. Still, poisonous fish gallbladder also causes ARF and induces damage to the heart, liver, and gastrointestinal tract. Thus, fish gallbladder may produce multiple organ dysfunction syndrome (MODS) with accompanying mortality [28,33]. Sodium Cyprinol sulfate is a highly potent toxin in the bile of grass carp, which can induce MODS [34]. However, cases of MODS were not observed in the present study. To date, no cases of poisoning due to consumption of goat gallbladder are available. The constraint to ingestion is the size of the goat gallbladder might cause difficulty swallowing and choking.

A limitation of this research is the use of whole bile without characterization of its components [11]. Small volumes of bile in single goat gallbladders were not sufficient for the entire study, and bile from several gallbladders was pooled. Composition of GB may vary among individual goats. Male goats of the same strain were used to minimalize such variation. Bile used in TCM to treat liver dysfunction likely varies. [15]. The make-up of bile will be crucial to determining the qualities needed for treating disease. Thus, the characterization of bile is the next in further development of GB as an antimalarial drug.

A new series of bile acid-based 1,2,4-trioxanes have been synthesized as possible antimalarial agents. These compounds have been assayed for antimalarial activity against multiple drug-resistant *Plasmodium yoelii* in Swiss mice. The 1,2,4-trioxanes were chosen to develop synthetic substitutes for artemisinin. This latter agent is isolated from *Artemisia annua* and owes its antimalarial activity to the 1,2,4-trioxane moiety. This moiety is active against both chloroquine-resistant and chloroquine-sensitive *Plasmodium* spp [34]. Human bile is suggested to be highly effective in treating malaria-type diseases in the ancient Chinese materia medica [14]. Bile acids have also been used for drug delivery to take advantage of their particular physicochemical properties and biocompatibility, and as drug absorption enhancers, for both drug solubilization and permeation [35]. Further, UDCA, a bile component, was used in a clinical trial with COVID-19 patients in the USA [36].

Mild toxicity was observed in the present study, yet GB was effective in treating malaria in mice. Unadulterated GB, GB100, suppressed parasitemia nearly as well as DHP. Consumption of GB in Indonesia involves swallowing gallbladder whole without further processing. Testimonial conversations with several consumers of bile suggested that fevers disappeared, mosquitoes did not bite, malarial infection regressed, and stamina increased after consuming GB. These individuals also reported no side effects (unpublished).

## Conclusion

GB might be developed as an antimalarial drug. The fluid suppressed parasitemia and did not cause liver or kidney dysfunction in uninfected mice. However, GB might cause toxicity if consumed too often. Such use should be avoided pending further investigation.

## Authors’ Contribution

HA: Research project leader, coordinator and designed the research, analyzed data, drafted manuscript, performed *P. berghei* ANKA infection and GB treatment. RRB: Originator of research ideas and consultant. WTH: Research initiator and counted the parasitemia. KAW and KNA: Sacrificed the mice, collected blood, and organized the data. PIBA: Statistical data analysis. PW: Clinical pathology analysis. All authors read and approved the final manuscript.
